# Spatial Transmission of Swine Vesicular Disease Virus in the 2006–2007 Epidemic in Lombardy

**DOI:** 10.1371/journal.pone.0062878

**Published:** 2013-05-07

**Authors:** Claudia Nassuato, Gert Jan Boender, Phaedra L. Eblé, Loris Alborali, Silvia Bellini, Thomas J. Hagenaars

**Affiliations:** 1 Istituto Zooprofilattico Sperimentale della Lombardia e dell’Emilia Romagna (IZSLER), Brescia, Italy; 2 Central Veterinary Institute of Wageningen UR (CVI), Lelystad, The Netherlands; INSERM & Universite Pierre et Marie Curie, France

## Abstract

In 2006 and 2007 pig farming in the region of Lombardy, in the north of Italy, was struck by an epidemic of Swine Vesicular Disease virus (SVDV). In fact this epidemic could be viewed as consisting of two sub-epidemics, as the reported outbreaks occurred in two separate time periods. These periods differed in terms of the provinces or municipalities that were affected and also in terms of the timing of implementation of movement restrictions. Here we use a simple mathematical model to analyse the epidemic data, quantifying between-farm transmission probability as a function of between-farm distance. The results show that the distance dependence of between-farm transmission differs between the two periods. In the first period transmission over relatively long distances occurred with higher probability than in the second period, reflecting the effect of movement restrictions in the second period. In the second period however, more intensive transmission occurred over relatively short distances. Our model analysis explains this in terms of the relatively high density of pig farms in the area most affected in this period, which exceeds a critical farm density for between-farm transmission. This latter result supports the rationale for the additional control measure taken in 2007 of pre-emptively culling farms in that area.

## Introduction

Swine Vesicular Disease (SVD) is a contagious disease of pigs caused by an Enterovirus of the Picornaviridae family. Often the disease is considered as a “pen disease” (as opposed to a farm disease), as typically morbidity strongly differs between pens, with some pens showing high morbidity [1]. It is hypothesized that the between-farm spreading routes of the disease include indirect contacts such as through contaminated lorries [1]. At the end of 2006 an outbreak of SVD was recorded in Italy in an area that had been free of SVDV since 2002. Epidemic spread of the disease occurred within the Italian northern regions, in particular in Lombardy where the areas affected were the most densely populated of the region. The SVD outbreaks reported in Lombardy could be grouped in two epidemic periods: the first one lasted from November 2006 to February 2007 (period 1), the second from May 2007 to October 2007 (period 2). In the area of the first period, transmission seemed to have been brought (locally) under control once control measures as prescribed by EU legislation, including movement restrictions, were implemented. One of the areas affected in period 2 was the most densely populated of the region (with more than 3000 pigs/km^2^) and despite the implementation of movement restrictions, epidemic spread could not be halted, leading to the decision to introduce pre-emptive depopulation of farms as an additional control measure in this area.

In this study we analyze and quantitatively characterize the pattern of SVDV spread in the two periods and areas, by quantifying the distance-dependent probability of between-farm transmission. Using the probability function obtained and the farm location data for the two areas, we are able to quantify the overall between-farm transmission risks in the two areas and periods. The parameter estimates thus obtained help elucidating the role of both farm density and movement restrictions in determining the pattern of spread. In particular, the analysis explains the observed differences in spreading patterns between the two periods in terms of the differences in the implementation of movement restrictions and the differences in (local) farm density in the areas affected.

**Figure 1:**
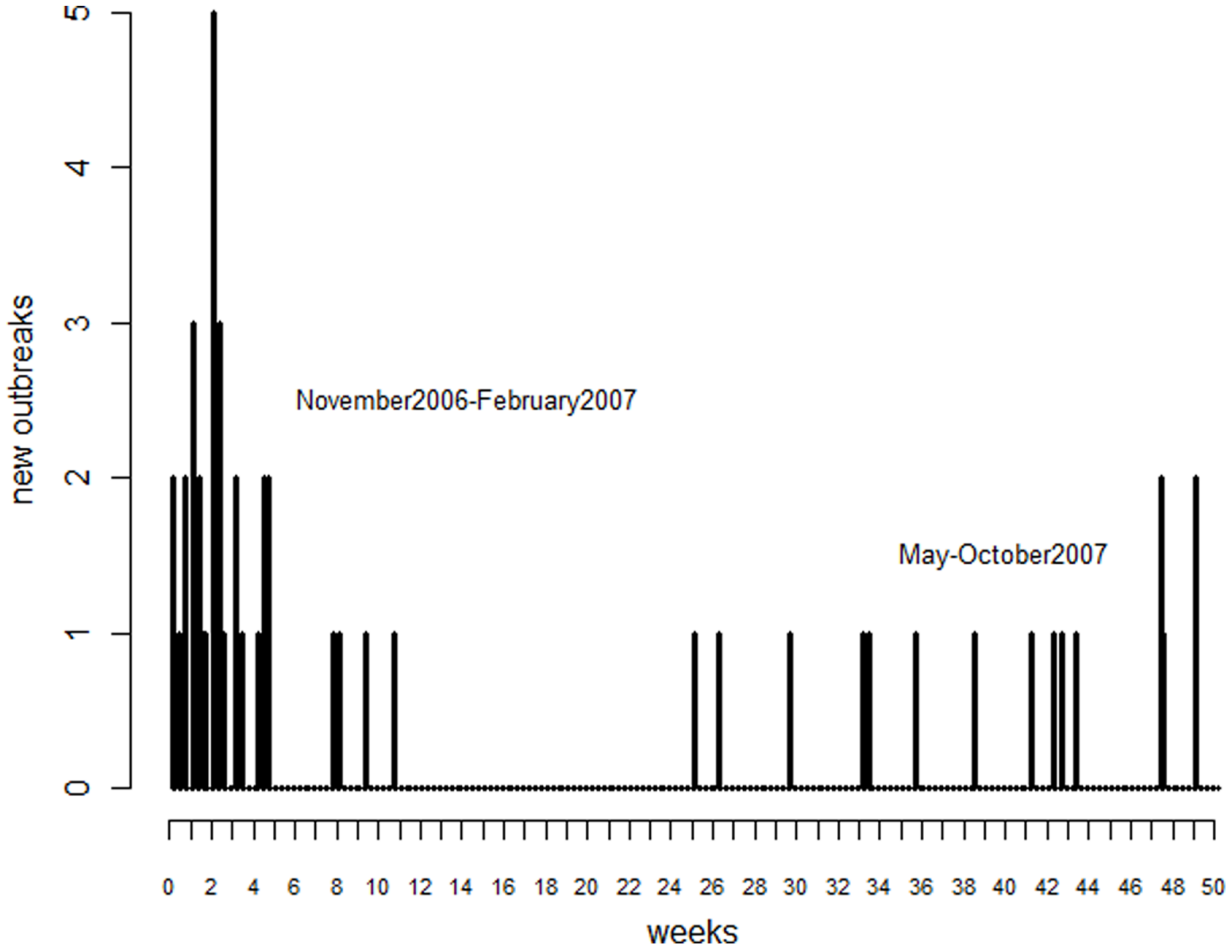
SVDV incidence through time. Weekly number of new SVDV outbreaks through time (14 November 2006 to 22 October 2007).

A descriptive overview of the epidemic spread and the chronology of control measures is given in [2]. Briefly, on 2 October 2006, after SVD had been absent from the region for four years, 11 pigs tested seropositive in a slaughterhouse in the province of Bergamo, which processed animals from various sources. Ultimately in November 2006 a pig dealer farm in the province of Verona (in the Veneto region) was found to be the source of the infection. Tracing pig movements from this farm, further outbreaks were identified in Veneto and also in Lombardy; in the latter region the disease spread for more than one year [2]. In February 2007, the outbreak seemed to have been controlled and eradication achieved. However, in May 2007, after a period without new outbreaks of about three months, the disease reappeared on a farm in Cremona, a province in the region of Lombardy that was not affected in the previous epidemic period. During 2006–2007, a total of 36 outbreaks were recorded in period 1 which lasted from November 2006 to February 2007 and 16 in period 2 which lasted from May 2007 to October 2007 (Figure 1).

**Figure 2:**
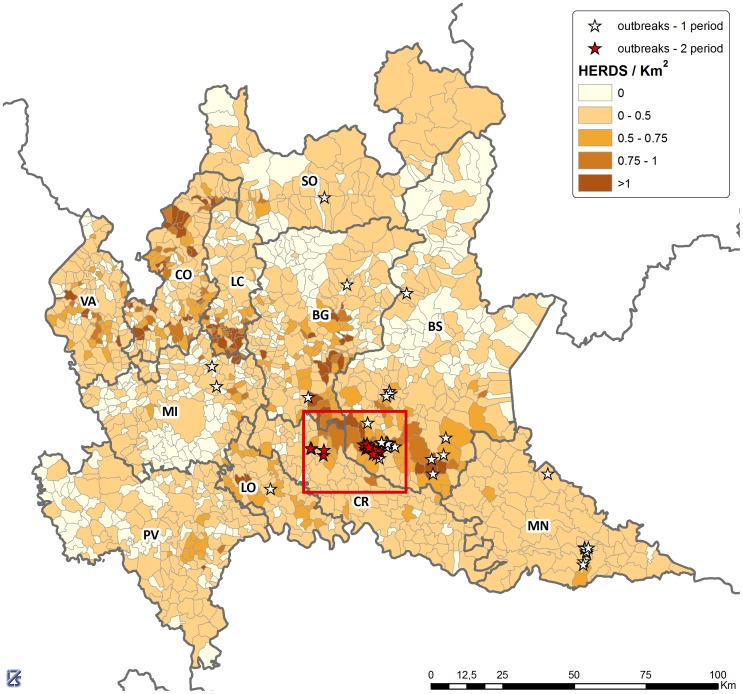
Outbreak locations. Map of Lombardy with the locations of the outbreaks arisen in the two epidemic periods (purple symbols: period 1, red symbols: period 2) and with municipalities color coded according to pig farm density (measured in herds per km^2^). The labels are referring to the provinces; BG: Bergamo, BS: Brescia, CO: Como, CR: Cremona, LC: Lecco, LO: Lodi, MN: Mantua, MI: Milan, PV: Pavia, SO: Sondrio, VA: Varese. We note that the average farm density across a whole municipality is generally lower than the actual local farm density around farm locations in that municipality.

**Figure 3:**
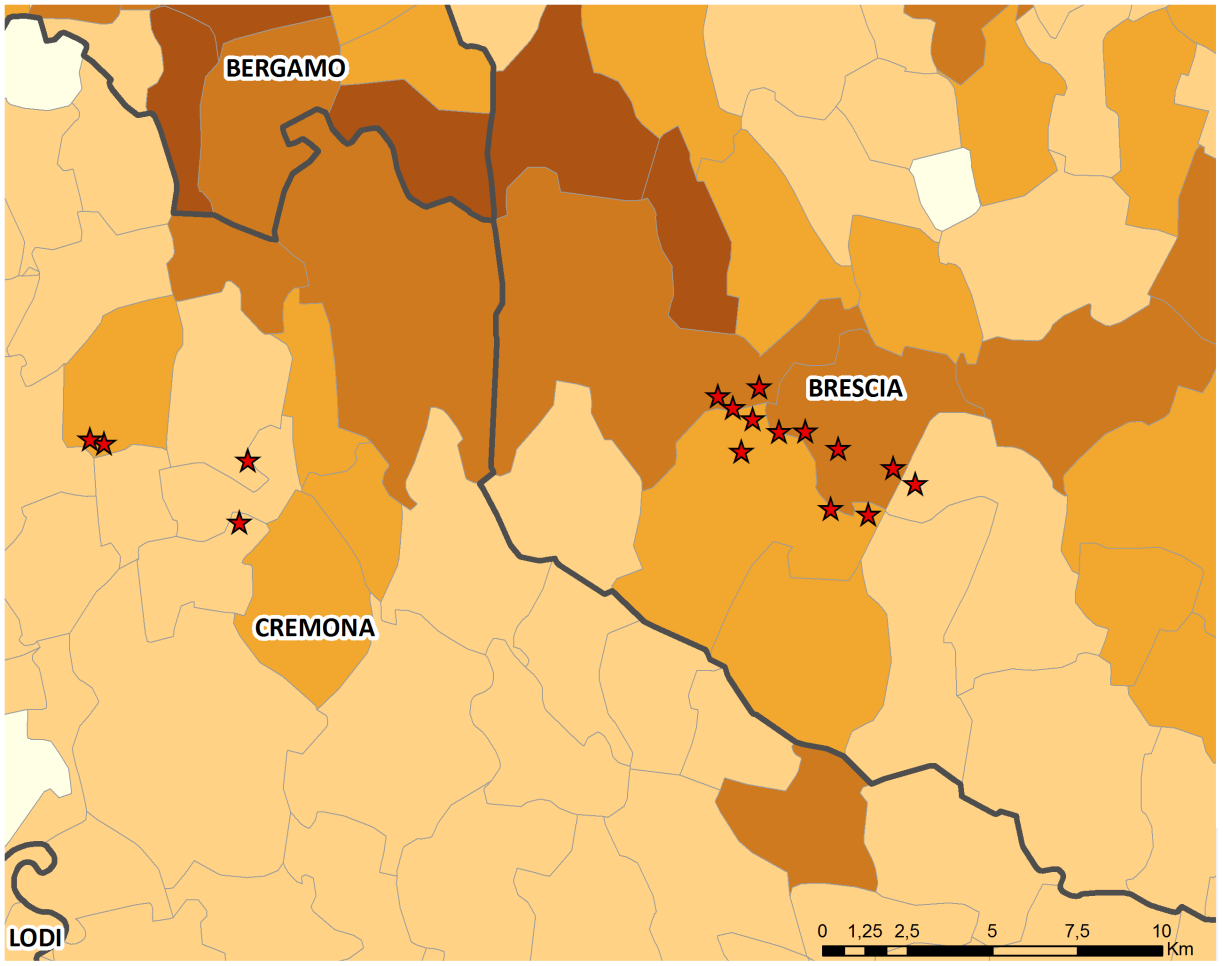
Outbreak locations in period 2. Detail of the areas affected by the epidemic in period 2. Across the affected area in Brescia that was pre-emptively depopulated, the local farm density is close to 1 farm per km^2^ (for details see Figure 5 of [2]).

Period 1 involved an extended area extending across much of the Lombardy region including the provinces of Brescia, Mantua, Bergamo, Lodi, Milan and Sondrio while period 2 involved the provinces of Cremona and Brescia (Figures 2 and 3).

The control measures followed European legislation established by Council Directive 92/119/EEC, Annex II [2], including the creation of protection (3 km) and surveillance (10 km) zones around outbreak farms as well as movement restrictions. The chronology of the movement restrictions applied in Lombardy is detailed in Tables S1 and S2. As can be seen from Tables S1 and S3, all except only three of the estimated dates of introduction during period 1 occurred before 17 November 2006, when transport movement restrictions were put in place. In contrast, during period 2 all 16 outbreaks except three have an estimated date of infection that is later than the last date of a series of cumulative movement restrictions (5 June). In this second period the outbreaks occurred in a limited area spanning across the border between the provinces of Brescia and Cremona. In the part in Brescia, with more than 3000 pigs/km^2^, SVD continued to spread despite control measures. In this area, in addition to the application of the control measures of Council Directive 92/119/EEC, it was decided to pre-emptively depopulate 15 farms (42,205 pigs) considered at risk of infection. Other holdings were located in the area but they were empty at the time of the epidemic [2].

## Materials and Methods

### Data

Our analysis requires the following two pieces of information [3]: the geographical locations of all farms that are at risk of SVDV infection, and an assessment of the infection status (susceptible, infected, infectious, removed) of each farm during the epidemic. The first piece was obtained from the regional veterinary database of Lombardy, maintained by the Veterinary Service. The second piece was obtained as follows. We assumed the infectious period of outbreak farms to be equal to the interval between the estimated date of virus introduction at a farm and the date of cleaning and disinfection of the premises. We identified farm removal from the pool of infectious farms (or from the pool of susceptible farms in case of pre-emptive culling) with the day of cleaning and desinfection of the depopulated premises. For each individual outbreak the date of virus introduction on a farm was estimated from laboratory test results and contact tracing data gathered during outbreak investigations (for a description of the contact tracing see [2]). It was defined as the date of contact in case of known contact with other outbreaks by animal movement or lorries for the transport of dead animals. Otherwise, it was estimated by adding 15, 20 and 30 days respectively to the date of blood sampling if the ELISA isotype-specific detected IgM only, both IgM and IgG, and IgG only, respectively, in accordance to the findings of an experimental infection reported in [4]. In case of virus isolation without antibodies the date of introduction of SVDV to the farm was set to 3 days before the date of withdrawal of blood samples. In 20 outbreaks of period 1 the date of infection was traced back to a contact that was considered likely to be the cause of transmission. In period 2 none of the outbreaks could be traced back. The estimated number of infectious farms through time, as well as the estimated number of newly infected farms through time, is given in Tables S3 and S4.

In line with the expected accuracy of the estimates for the dates of virus introduction, the analysis was carried out using time steps of one week (see below), i.e. all dates were rounded to weekly time points. The one-week time resolution is also motivated by our model assumption to neglect any period in which farms are infected but not yet infectious. Starting from the detailed date estimates, infection dates falling within a given week were attributed to transmission in that week. Likewise, starting from the detailed culling dates, the status of all farms was updated at the end of each week.

Whereas the official number of outbreaks in period 1 is 36, we work with 34 outbreaks in the analysis. This is because two pairs of outbreaks had to be interpreted in the analysis as single outbreaks, as (in both cases) both farms had the same location and where part of the same epidemiological unit. The earliest estimated date of introduction of the two was taken as date of introduction.

We denote the mean infectious period across outbreak farms by 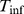. As a measure of variation of the infectious period between outbreak farms we use the coefficient of variation, denoted by CV.

### Method of Analysis

We adopt the approach developed in [3], estimating the infection hazard posed by a single infectious farm to a susceptible farm as a function of the straight-line distance between the two farms. This infection hazard is mathematically denoted as 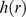, with *r* being the between-farm distance, and will be referred to as the between–farm transmission kernel from now on. This kernel is a statistical model description of the average total infection hazard between an infectious and a susceptible pig farm a distance *r* apart, across all possible transmission routes. We here estimate the between-farm transmission kernel separately for each of the two periods in the epidemic, using the following parameterization:
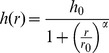



Here 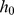 is the transmission hazard at very short distances; it gives the maximum value for 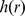, which is attained at 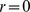. The remaining two parameters 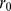 and 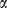 together determine the shape of the 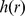 curve. The parameter 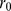 is a scaling distance, i.e. it determines on which distance scale the transmission hazard is declining to lower values; 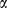 is a parameter characterizing the long-distance shape of 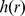, i.e. it determines how fast the hazard declines with increasing distance *r* at long distances. This parameterization limits the number of estimable parameters to three, whilst still being general enough to include a wide range of possible shapes. Amongst a set of alternative parameterizations, it was found to provide the best model fit to the between-farm transmission patterns of Highly Pathogenic Avian Influenza in Dutch poultry analyzed in [3]. The same parameterization has also been used for analyses of Foot-and-Mouth Disease and Bluetongue transmission in Dutch ruminants [5], [6], and for Highly Pathogenic Avian Influenza in Italian poultry [7]. We use this form not only because it performed best in the previous analysis on a different virus, but also because it allows a direct comparison of the longer-distance scaling behaviour of between-transmission between the two periods, through comparing the estimates for the parameter 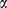. As we have two different periods, we will estimate six parameters in total: 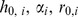 (*i* = 1,2). Below we will drop the index *i* to these parameters as in the Results section it will always be clear from the context which one of the two periods is considered.

As is common for the type of analysis carried out (see also [5]–[7]) we use the approximation to work with farms as individual epidemiological units, and we are assuming constant infectivity of farms between date of infection and date of culling. The rationale for not modeling individual animals is that we are here interested in characterizing between-farm transmission; for this purpose there a description on the level of the individual animal would not provide any benefits, but only introduce additional uncertain parameters.

We estimate the transmission kernel parameters using Maximum-Likelihood estimation, obtaining univariate confidence bounds using the likelihood ratio test. This analysis was carried out using time steps of one week. The model likelihood is then given by the product of the binomial probabilities of all the week-by-week events: each farm that is still susceptible at the beginning of the week either escapes from infection, or becomes infected by any of the infectious farms present that week:
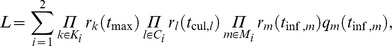
where the set 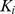 contains all farms that remained uninfected up until the end of the epidemic period *i* (denoted by 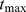) and that were not culled, 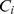 contains the farms that were not infected but that were culled (at times 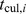) in period *i*, and 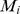 contains the farms that were infected (at times 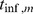) in period *i* except for the first infected farm of the period. Further, 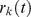 denotes the probability that farm *_k_* remains uninfected up to day *t*,



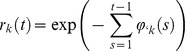
and 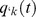 denotes the probability that a hitherto susceptible farm *k* is infected on day *t*:



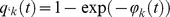



Where 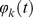 is the force of infection on a susceptible farm *k*:
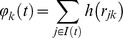



With 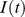 the set of all infectious farms at time *t*, and 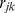 the straight-line distance between farms *k* and *j*.

As explained in [3], a critical farm density 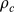, above which epidemic between-farm spread can occur, can be calculated using the estimated transmission kernel. This critical farm density can be used in a rule-of-thumb fashion as a critical value for the local farm density around farm locations, provided that the radius of the “local” area across which the local farm density is computed is comparable to the range spanned by the transmission kernel. For the kernel parameter regime relevant in this paper, 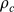 is in good approximation given by:
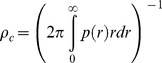
where 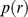 is the probability that an uninfected farm will be infected by an infected farm a distance *r* away. Here we calculate 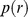 approximately using the mean infectious period 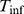:



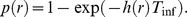



The integral in the above expression for 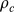 is only finite if the parameter 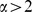. For 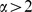 it is infinite, i.e. the critical farm density 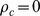. The epidemiological interpretation of this mathematical observation is that for 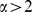 the farms at very long distances contribute only marginally to the total risk of transmission even though there are many more farms at long than at short and intermediate distances [3]. In this regime, the total transmission risk (as measured by the basic reproduction number, see [3]) is finite even if farm densities would not decline at large distances, because there is a limitation on the spatial range of between-farm transmission determined by 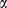: the higher 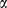 the shorter the range. This limited range is intrinsic to the transmission process(es) characterized by 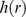. In contrast, for 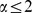 farms at very long distances do contribute substantially to the total risk of transmission. Thus, in this regime the finite total risk of transmission is not due to an intrinsically finite spatial range of transmission, but only due to limitations in the spatial extent of the host/farm population, arising e.g. due to geographical and economic constraints.

We note that the transmission kernel is a measure of the transmission risk from an infectious to a given susceptible farm at a given distance. In order to further characterize the transmission pattern, it is useful to also define the average distance 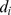 over which transmission takes place within a given epidemic period *i*. This quantity is calculated as a weighted average over distances 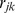 between possible “parent-offspring” pairs of outbreak farms (*j, k*), with the estimated kernel 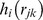 as weighting factor:
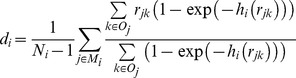



Here 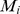 is the number of outbreak farms in period *i* and 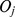 is the set of all candidate parent outbreak farms for offspring outbreak farm *j*, i.e. those that were infectious in the week that farm *j* was estimated to become infected. The average is over 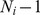 farms as for the first infected farm of a period there are no candidate parent outbreak farms in the same period.The analysis has been coded and executed using both the R [8] and Mathematica [9] software, yielding the same results.

## Results

For period 1 the estimate for the parameter 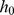 was 0.02 (95% CI: 0.005–0.22) per week, for the parameter 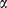 it was 1.84 (1.47–2.21) and for parameter 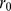 it was 520 (80–1560) m. In this period the mean infectious period of the outbreak farms 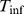 was equal to 7.8 weeks, with CV equal to 0.3. For period 2 the estimate for the parameter 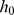 was 0.03 (0.009–0.3) per week, for the parameter 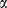 it was 2.4 (1.85–3.1) and for parameter 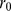 it was 1050 (210–2240) m. In this period the mean infectious period 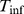 was 3.1 weeks, with CV equal to 0.3. The estimated numbers of newly infected and infectious farms by week, on which our parameter estimates are based, can be found in Tables S3 (period 1) and S4 (period 2).

**Figure 4:**
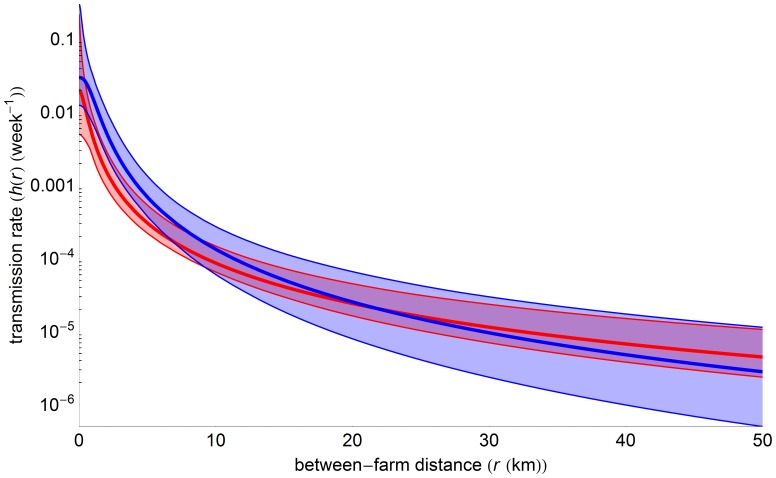
Transmission kernel comparison. Comparison between the estimated transmission kernels for period 1 (red) and period 2 (blue). Thick lines: Maximum-Likelihood estimate of the transmission kernel; thin lines: confidence bounds calculated as explained in [3].

The corresponding transmission kernels for the two epidemic periods are shown in Figure 4. The comparison shows in particular how the difference between the periods in the estimated value for the parameter 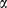 translates into a different long-distance (say *r* ≥30 km) behavior of the kernel: The higher point estimate for 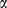
for period 2 produces a faster decline with distance. The differences in the estimates for the other two kernel parameters have their main effect at short and intermediate distances, causing the transmission kernel of period 2 to exceed that of period 1 for distances below approximately 22 km. When calculating the critical farm density 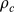 from the kernels estimated for the two periods, we find 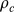 = 0.0 (0.–0.31) farms/km^2^ for period 1 and 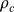 = 0.59 (0.0–1.10) farms/km^2^ for period 2. Finally, for the average distance of transmission in the two periods we find 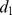 = 18.17 (17.10–19.56) km and 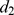 = 7.92 (7.90–7.96) km.

## Discussion

The epidemic of 2006–2007 SVDV epidemic in Italy consisted of two sub-epidemics, differing in terms of the provinces or municipalities that were affected and also in terms of the timing of implementation of movement restrictions. The difference between these two periods in the mean estimated infectious period of the outbreak farms 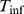 (7.8 weeks in period 1 versus 3.1 weeks in period 2) is striking, and shows that in period 2 outbreaks were on average detected much earlier than in period 1. The experience of period 1 has likely helped the improved detection in period 2 e.g. through increased awareness. By quantifying between-farm transmission probabilities as a function of between-farm distance, we also found that the spatial transmission characteristics differ between the two periods. In particular we found that the average distance of transmission differs significantly between the two periods (non-overlapping confidence intervals), 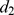 being smaller than 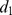. From the model viewpoint the average distance of transmission is determined by both the transmission kernel and the farm density pattern in the affected area. Therefore, the difference found between 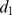 and 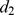 can be understood as being in part due to a difference in the transmission kernel (i.e. the kernel parameters) between the two periods (where the difference in the kernel parameters is not statistically significant itself). Based on the point estimates for the kernel parameters, the probabilities of transmission over relatively long distances were found to be lower in the second than in the first period, indicating that movement restrictions in the second period had an effect. This interpretation is in line with the observation, from Tables S1 and S3, that most of the estimated dates of introduction during period 1 occurred before movement restrictions were put in place (17 November 2006). In contrast, in period 2 all 16 outbreaks except three have an estimated date of infection that is later than the last date of a series of cumulative movement restrictions (5 June 2007). The observation that for 20 out of 34 outbreaks in period 1 a likely route was traced in terms of an animal transport suggests that transport restrictions make an important contribution to reducing the transmission risks. Indeed, only a minority of three outbreaks were estimated to have been caused after the moment that the first movement restrictions had been put in place. In period 2 however, more intensive transmission occurred over relatively short distances. Our model analysis explains this mainly in terms of the relatively high farm density of the area most affected in this period, which exceeds a critical farm density for between-farm transmission.

The shape of the transmission kernel for relatively large distances is determined by 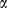 and thereby our estimates for 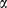
are particularly informative to compare the transmission in period 1 and 2. We note that for period 1 the point estimate for 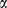 is below 2, corresponding to a critical farm density 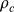 of 0 farms/km^2^, as explained in the methods. This means that in period 1 there is both local and longer-range transmission, and the total transmission risks across all ranges are such that epidemic spread is expected as soon as the farm density is non-zero. In other words, the total between-farm transmission in period 1 is only limited through limitations in the spatial extent of the host/farm population. In contrast, for period 2 the point estimate for 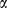 is above 2, which means that there is a non-zero critical farm density 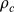. The fact that the epidemic was still not under control in Brescia in period 2 despite the movement restrictions is explained by our analysis in terms of the high density of farms in the area. Indeed, with a density close to 1 farm per km^2^, the density in this area exceeds the point estimate of 0.59 farms/km^2^ for the critical density 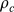. This result supports the rationale for the pre-emptive culling adopted as an additional control measure taken in this area in 2007.

Our analysis yields better insight in the spatial spread of SVDV in Lombardy during the 2006–2007 epidemic. In particular, the analysis helped in quantifying and comparing the distance-dependent transmission characteristic of the infection between the two main periods of the epidemic. The difference found between the two periods in the point estimate of the transmission kernel parameter 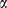 suggests that movement restrictions mostly reduced the transmission between farms that are relatively long distances apart. In high-farm-density areas, where transmission over relatively short distances is promoted by the presence of a high number of farms, such movement restrictions may not be sufficient to control the between-farm spread, as is exemplified by period 2 of the epidemic. This result is in close correspondence with the findings for avian influenza transmission between poultry in The Netherlands, where high farm density areas were shown to be high-risk areas for between-farm spread [3].

## Supporting Information

Table S1 **Chronology of movement restrictions in Lombardy in period 1.**
(DOC)

Table S2 **Chronology of movement restrictions and preventive culling measures in period 2.**
(DOC)

Table S3 **Number of newly infected and infectious farms per week for period 1.**
(DOC)

Table S4 **Number of new infected and infectious farms per week for period 2.**
(DOC)
