# Supplementary material for: Spatial Transmission of Swine Vesicular Disease Virus in the 2006–2007 Epidemic in Lombardy
Source: PLoS One. 2013 May 7;8(5):e62878. doi: 10.1371/journal.pone.0062878 (PMC3647039; doi:10.1371/journal.pone.0062878)
Supplement: Table S1 — Chronology of movement restrictions in Lombardy in period 1. (DOC) [file pone.0062878.s001.doc]

Table S1. Chronology of movement restrictions in Lombardy in period 1.

| **Date** | **Event** |
| --- | --- |
| 2 October 2006 | Serologically positive animals at slaughterhouse in the province of Bergamo |
| 14 November 2006 | First outbreak officially notified in the Bergamo province |
| 17 November 2006 | Provinces of Bergamo and Brescia placed under restrictions* |
| 30 November 2006 | Provinces of Mantua and Sondrio placed under restrictions* |
| 28 December 2006 | Province of Lodi placed under restrictions* |
| 2 February 2007 | Province of Milan placed under restrictions* |
| 2 February 2007 | Restrictions lifted in the province of Bergamo (no outbreak detected in the province since the 1 December 2006) |
| 20 February 2007 | Restrictions lifted in the provinces of Brescia, Sondrio and Lodi (no outbreak detected in these provinces since the 11 January 2007) |
| 23 March 2007 | Restrictions lifted in the province of Milan (no outbreak detected in the province since the 7 February 2007) |

*For all pig holdings the disease-free status was suspended, and animal movements were banned. Subsequently, animal movements were re-allowed from herds re-acquiring of the disease-free status after testing negative, with re-testing every 28 days for maintenance of status.
