# Supplementary material for: Spatial Transmission of Swine Vesicular Disease Virus in the 2006–2007 Epidemic in Lombardy
Source: PLoS One. 2013 May 7;8(5):e62878. doi: 10.1371/journal.pone.0062878 (PMC3647039; doi:10.1371/journal.pone.0062878)
Supplement: Table S2 — Chronology of movement restrictions and preventive culling measures in period 2. (DOC) [file pone.0062878.s002.doc]

**Table S2**. **Chronology of movement restrictions and preventive culling measures in period 2.**

| **Date** | **Event** |
| --- | --- |
| 7 May 2007 | First confirmed outbreak located in the province of Cremona |
| 9 May 2007 | Province of Cremona placed under restrictions* |
| 5 June 2007 | Whole region of Lombardy placed under restrictions* |
| 22 October 2007 | Last recorded outbreak |
| 9 November 2007 | Preventive depopulation in part of Brescia |

*For all pig holdings the disease-free status was suspended, and animal movements were banned. Subsequently, animal movements were re-allowed from herds re-acquiring of the disease-free status after testing negative, with re-testing every 28 days for maintenance of status.
