# Supplementary material for: Spatial Transmission of Swine Vesicular Disease Virus in the 2006–2007 Epidemic in Lombardy
Source: PLoS One. 2013 May 7;8(5):e62878. doi: 10.1371/journal.pone.0062878 (PMC3647039; doi:10.1371/journal.pone.0062878)
Supplement: Table S3 — Number of newly infected and infectious farms per week for period 1. (DOC) [file pone.0062878.s003.doc]

**Table S3. Number of newly infected and infectious farms per week for period 1.**

| **Week** | **Newly infected^a^** | **Infectious^b^** |
| --- | --- | --- |
| 22 Sep 2006 | 6 | 0 |
| 29 Sep 2006 | 2 | 6 |
| 06 Oct 2006 | 4 | 8 |
| 13 Oct 2006 | 2 | 12 |
| 20 Oct 2006 | 4 | 14 |
| 27 Oct 2006 | 0 | 18 |
| 02 Nov 2006 | 4 | 18 |
| 09 Nov 2006 | 8 | 22 |
| 16 Nov 2006 | 1 | 30 |
| 23 Nov 2006 | 0 | 30 |
| 30 Nov 2006 | 0 | 30 |
| 07 Dec 2006 | 1 | 22 |
| 14 Dec 2006 | 1 | 22 |
| 21 Dec 2006 | 0 | 11 |
| 28 Dec 2006 | 1 | 7 |
| 04 Jan 2007 | 0 | 7 |
| 11 Jan 2007 | 0 | 4 |
| 18 Jan 2007 | 0 | 3 |
| 25 Jan 2007 | 0 | 2 |
| 01 Feb 2007 | 0 | 2 |
| 08 Feb 2007 | 0 | 1 |
| 15 Feb 2007 | 0 | 1 |

^a^number of newly infected farms for each week of the epidemic, as calculated from the estimated dates of virus introduction onto the outbreak farms.

^b^number of infectious farms, as estimated from the outbreak data, present at the corresponding week of the epidemic. NB: Two sets of two outbreaks in the province of Mantua were each grouped together because the herds could be considered as part of the same epidemiological unit.
