# Supplementary material for: Spatial Transmission of Swine Vesicular Disease Virus in the 2006–2007 Epidemic in Lombardy
Source: PLoS One. 2013 May 7;8(5):e62878. doi: 10.1371/journal.pone.0062878 (PMC3647039; doi:10.1371/journal.pone.0062878)
Supplement: Table S4 — Number of new infected and infectious farms per week for period 2. (DOC) [file pone.0062878.s004.doc]

**Table S4. Number of new infected and infectious farms per week for period 2.**

| **Week** | **Newly infected^a^** | **Infectious^b^** |
| --- | --- | --- |
| 20 Apr 2007 | 1 | 0 |
| 27 Apr 2007 | 0 | 1 |
| 04 May 2007 | 1 | 1 |
| 11 May 2007 | 0 | 2 |
| 18 May 2007 | 0 | 0 |
| 25 May 2007 | 1 | 0 |
| 01 Jun 2007 | 0 | 1 |
| 08 Jun 2007 | 0 | 1 |
| 15 Jun 2007 | 2 | 1 |
| 22 Jun 2007 | 0 | 2 |
| 29 Jun 2007 | 1 | 2 |
| 06 Jul 2007 | 0 | 3 |
| 13 Jul 2007 | 0 | 1 |
| 20 Jul 2007 | 1 | 1 |
| 27 Jul 2007 | 0 | 1 |
| 03 Aug 2007 | 0 | 1 |
| 10 Aug 2007 | 1 | 1 |
| 17 Aug 2007 | 1 | 2 |
| 24 Aug 2007 | 1 | 2 |
| 31 Aug 2007 | 1 | 3 |
| 07 Sep 2007 | 0 | 3 |
| 14 Sep 2007 | 1 | 1 |
| 21 Sep 2007 | 2 | 2 |
| 28 Sep 2007 | 0 | 3 |
| 05 Oct 2007 | 2 | 3 |
| 12 Oct 2007 | 0 | 5 |
| 19 Oct 2007 | 0 | 5 |
| 26 Oct 2007 | 0 | 2 |

^a^number of new infected farms, as estimated from the outbreak data, for each week of the epidemic.

^b^number of infectious farms, as estimated from the outbreak data, present at the corresponding week of the epidemic.
